# Development and validation of the hospice professional coping scale among Chinese nurses

**DOI:** 10.1186/s12913-024-10970-9

**Published:** 2024-04-20

**Authors:** Yanting Zhang, Li Zheng, Yanling He, Min Han, Yu Wang, Jinyu Xv, Hui Qiu, Liu Yang

**Affiliations:** 1 Department of Critical Care Medicine, Hubei Clinical Research Center for Critical Care Medicine, Zhongnan Hospital of Wuhan University, Wuhan, Hubei 430071 China; 2 Department of Lung Cancer Radiotherapy and Chemotherapy, Zhongnan Hospital of Wuhan University, Wuhan, Hubei 430071 China; 3 Department of Gynecological Tumor Radiotherapy and Chemotherapy, Zhongnan Hospital of Wuhan University, Wuhan, Hubei 430071 China

**Keywords:** Hospice care, End-of-life patients, Care burden, Reliability, Validity, Scale research

## Abstract

**Background:**

Hospice care professionals often experience trauma patient deaths and multiple patient deaths in a short period of time (more so than other nurses). This repeated exposure to the death process and the death of patients leads to greater psychological pressure on hospice care professionals. But at present, people pay more attention to the feelings and care burden of the family members of dying patients but pay less attention to medical staff. Thus, this study aimed to develop a scale on the burden of care for hospice care providers and assess the coping capacity of hospice professionals. Raising awareness of the psychological burden of hospice professionals.

**Methods:**

Through a literature review, research group discussion, Delphi method and a pre-survey of professional coping skills among nurses, 200 hospice professionals who had received training in hospice care from pilot institutions engaged in or providing hospice care were selected for investigation. Cronbach’s α coefficient and split-half reliability were used to test the internal consistency of the scale, and content validity and explore factor analysis (EFA) were used to test the construct validity of the scale.

**Results:**

Two rounds of Delphi methods were carried out, and the effective recovery rate was 100%. The expert authority coefficients of the two rounds were 0.838 and 0.833, respectively. The Kendall’s W coefficient of experts in the first round was 0.121 ~ 0.200 (*P* < 0.05), and the Kendall’s W coefficient of the second round was 0.115–0.136 (*P* < 0.05), indicating a good level of expert coordination. The final survey scale for the care burden of hospice professionals included four dimensions—working environment (9 items), professional roles (8 items), clinical nursing (9 items) and psychological burden (7 items)—with a total of 33 items. The total Cronbach’s α coefficient of the scale was 0.963, and the Cronbach’s α coefficients of the working environment, professional roles, clinical nursing and psychological burden dimensions were 0.920, 0.889, 0.936 and 0.910, respectively. The total split-half reliability of the scale was 0.927, and the split-half reliability of each dimension was 0.846, 0.817, 0.891, and 0.832. The content validity of the scale items ranged from 0.90 to 1.00. Exploratory factor analysis revealed 5 common factors, with a total cumulative contribution rate of 68.878%. The common degree of each item in the scale was > 0.4, and the factor loading of each item was also > 0.4.

**Conclusion:**

The scale is an open-access, short, easy-to-administer scale. And which for assessing hospice care burden among hospice professionals developed in this study demonstrated strong reliability and validity. This tool can serve as a dependable instrument for evaluating the burden of hospice care for terminally ill patients by professionals in the hospice setting.

**Supplementary Information:**

The online version contains supplementary material available at 10.1186/s12913-024-10970-9.

## Background

Hospice care refers to providing patients with terminal diseases with physical, psychological, and spiritual care, as well as humanistic care, by controlling the symptoms of pain and discomfort to improve their quality of life and help them die comfortably, calmly, and with dignity [1]. In June 2020, hospice care was incorporated into Chinese law for the first time. Article 36 of the Law on the Promotion of Basic Medicine and Health clearly stipulates that medical institutions provide hospice care and other medical and health services to citizens [2]. As early as 2016, the National Health and Family Planning Commission issued the National Nursing Development Plan (2016–2020) [3], noting the need to strengthen capacity-building for hospice care and improve relevant mechanisms. While the state vigorously promoted the development of hospice care, it also exposed many problems. These problems include the relatively traditional concept of death for our citizens, uneven development in the field of hospice care, and a lack of human resources and teams. The legal provisions on hospice care are relatively broad, and a lack of understanding of hospice care services can easily lead to medical disputes [4, 5]. This not only poses numerous obstacles to the practical development of hospice care but also exposes hospice nursing staff to complex clinical situations [6].

According to previous studies, hospice care professionals often experience traumatic patient deaths and multiple patient deaths in a short period of time (more so than other nurses) [7, 8]. This repeated exposure to the death process and the death of patients leads to greater psychological pressure on hospice care professionals [9, 10]. In different groups, social support alleviates many adverse outcomes of hospice care professionals, such as high psychological stress and high emotional burnout [11, 12]. In addition, nurses in oncology departments and palliative care departments need to continue to provide empathy and care for patients, not only to bear psychological pressure but also to undertake the emotional work of patients’ families, which easily results in empathy fatigue [13, 14]. The psychological stress caused by empathy fatigue seriously affects the mental health and nurse‒patient relationships of nurses and may even lead to their resignation [15]. The assessment of the care burden of hospice care professionals can provide a reference for the formulation of relevant policies, provide guidance for terminally ill patients and their families to implement better hospice care services, provide comfort and respect for people in the final stages of life, and promote the development of hospice care [16].

At present, people pay more attention to the feelings and care burden of the family members of dying patients but pay less attention to medical staff. In addition, the related assessment tools in China are mainly aimed at assessing nurses’ knowledge, attitudes and behaviors related to hospice care. For example, the assessment tool used by Zheng is the self-developed hospice care attitude scale [17, 18], and few studies have assessed the psychological stress of medical staff. However, due to cultural differences, assessment tools such as the Zarit Nursing Burden Scale (ZBI) [19] are not applicable in other countries. In recent years, some scholars have developed and verified self-care ability assessment tools for hospice care practitioners, but there is still a lack of assessments of care burden [20, 21]. Therefore, this study provides a tool for assessing the care burden level of hospice care professionals by developing a scale for hospice care professionals and testing its reliability and validity. In addition, this study provides a clearer understanding of the current situation and influencing factors of hospice care burden in China and evaluates the effectiveness of interventions to reduce hospice care burden.

## Methods

### Development and procedure

#### Constructing a scale item pool

Under the guidance of the Zarit Nursing Burden Scale (ZBI) [19], which uses hospice care/hospice care/health care personnel/nurses care/stress/empathy/psychological burden/fatigue as the key words, a large number of related studies were consulted through Pubmed/Web of Science/CINAL/China Knowledge Network/Wan Fang and other databases. To form a pool of items in the nursing burden scale for hospice care staff. The scale pool consists of 32 items, including working environment, professional role, clinical nursing and psychological burden. All the items were scored on a 5-point Likert scale, and they were all positive.

### Delphi method

#### Expert inclusion criteria

Bachelor’s degree or above; intermediate or above professional title; engaged in clinical work ≥ 5 years; were familiar with hospice care treatment and highly enthusiastic about this study; and voluntarily participated in and completed multiple rounds of inquiries.

#### Delphi method expert consultation form

The expert consultation form consisted of four parts: an introduction, basic information from the experts, a nursing burden scale for hospice care professionals, and an expert authority scale. The preface introduces the purpose, significance, and instructions of this survey. The basic expert information table includes age, sex, educational background, professional title, clinical working years, research field, and whether he or she is a graduate tutor. The nursing burden scale of hospice care professionals includes four dimensions: working environment, professional role, clinical nursing, and psychological burden. The importance and relevance of the scale items were evaluated by experts. For items that need to be modified, deleted, or added, experts can write down their comments in the corresponding “modified comments” column. Importance is divided into 5 levels: level 5 is highly important, level 1 is highly unimportant, relevance is divided into 4 levels, level 4 is highly relevant, and level 1 is highly irrelevant.

The expert authority scale includes the degree of experts’ familiarity with hospice care (very familiar = 1, relatively familiar = 0.8, generally familiar = 0.6, unfamiliar = 0.4, very unfamiliar = 0.2) and the influence of judgment basis (work experience judgment/theoretical knowledge analysis/domestic and foreign relevant data) on expert judgment.

#### Distribution and recycling of scales

During the first round of Delphi, the items of the scale were made into an expert consultation form and sent to all the experts by email. The experts were invited to provide responses within a week and to integrate, analyse and discuss their views. After an interval of 2 weeks, the second round of the credit scale is sent to all the experts via the same process as the first round. The selection criteria for the items were as follows: mean importance ≥ 4, coefficient of variation (CV) ≤ 0.25, and full score ratio > 0.20. Items that met all three criteria were retained. If only 1–2 criteria are met, further confirmation or panel discussion with the expert is required to decide whether to retain the criterion, and if none of the three criteria are met, the criterion is deleted [22].

#### Item modification content

After the first round of Delphi method, the items were added or modified according to the experts’ scores on the importance and relevance of the items as well as the expert’s advice. Three items with a coefficient of variation > 0.25 and a full score ratio < 0.2 were excluded (see supplement 1: Tables 1 and 2 for specific results). In the clinical nursing dimension, there is an item that does not meet the above criteria: “Do you think the terminally ill patients or their families you care for will require too much care for you?” After discussion with the working group, this item was retained because of its importance. The languages of 10 items had to be revised. One new item was added to each of the three dimensions of working environment, professional role and clinical nursing, and the new item was “Do you think that hospice care currently lacks the support of social recognition and other social forces?”, “Do you think it is more difficult for hospice workers to gain a sense of professional achievement?”, “Do you think that family members’ recognition and compatibility with hospice care is an important factor in carrying out work?”

After the second round of Delphi method, only one of the items in the clinical nursing dimension was modified: “strong death identity” was replaced by “patients who are pessimistic about death”. Finally, the nursing burden survey scale of hospice care professionals was developed, which included working environment (9 items), professional role (8 items), clinical nursing (9 items) and psychological burden (7 items), for a total of 33 items.

#### Pre-investigation

Using a convenience sampling method, 50 hospice care professionals who were engaged in or who received hospice care training in pilot hospice care institutions were selected as the research subjects in October 2022. In the course of the survey, the participants were closely observed for difficulty in understanding the scale and their opinions. After the last 2 rounds of Delphi method, all the entries were retained for formal investigation.

### Sample size

According to the rough estimation method of sample size proposed by clinical epidemiology, the sample size is 5 ∼ 10 times the number of items in the scale [23], and the final number of items in this scale is 33, so the sample size is 165 ∼ 330.

### Characteristics of participants

Using a convenience sampling method, 200 hospice care professionals who were engaged in or who received hospice care training in several hospitals or hospice pilot institutions were selected in December 2022, of which 150 were used for supplementary investigation. It should be noted that the supplementary survey objects here are the sample sizes collected after the presurvey. The inclusion criteria for participants were medical staff who participated in hospice care and who had received training, were aged ≥ 18 years, were clearly conscious, had good expression, provided informed consent, and had more than 2 years of work experience. The exclusion criteria were working for ≤ 2 years; not providing informed consent; only professionals who understood but did not participate in the hospice care system; and who had received training in the hospice care system.

### Survey tools

① The general and basic conditions of hospice care and nursing staff. ② The scale of care burden of hospice nurses included four dimensions: working environment (9 items), professional role (8 items), clinical nursing (9 items) and psychological burden (7 items). On a 5-point Likert scale, 1 indicates complete lack (very disagree), and 5 indicates proficiency (very much agree).

### Investigation procedure

The scale survey method was as follows: To ensure the smooth progress of the study, informed consent was obtained from the respondents before the scale survey, and the purpose and significance of this study were explained to the respondents to obtain cooperation. All the scales distributed in this study were distributed and completed through the scale stars. It can only be submitted after answering the set questions. It can only answer each time to ensure the rigor, authenticity and completeness of the scale. The scale collected must be reviewed by the research team, and if all the answers are the same, it will be determined to be invalid. A total of 250 copies were distributed in this study, and 200 copies were recovered.

### Statistical methods

The data were inputted by two people using EpiData 3.0 software, and SPSS 23.0 statistical software was used for descriptive analysis, project analysis, exploratory factor analysis [24], correlation analysis, reliability and validity testing. The specific contents of the analysis were as follows: the items of the scale were screened by the differentiation method, and the items were sorted according to their scores. The first 27% of the scores are high, and the remaining 27% are low. Then, the average score of each item was calculated for the high score and low score groups. Using the independent sample t test, if the average score of an item has no significant difference between the high score and the low score (0.05), the importance and differentiation of the item are not significant, and the entry is excluded [22]. Cronbach’s α coefficient and the Spearman Brown method were used to test the reliability. Content validity and construct validity were used to test the validity of the scale, item-level content validity (I-CVI) and average scale-level content validity (S-CVI/Ave) were used as content validity indicators, and exploratory factor analysis was used to determine the number of common factors, cumulative contribution rate and eigenvalues of the scale. The screening criteria for each item were cumulative contribution rate > 60%, eigenvalue > 1, common variance > 0.4, and factor load > 0.4 for each entry.

### Ethical considerations

All participants provided signed informed consent when reliability and validity tests were conducted. This study was approved by the Ethics Committee of Zhongnan Hospital of Wuhan University [2,022,119 K].

## Results

### Basic characteristics of the experts

A total of 20 experts were selected for this study, and the details are shown in Table 1.

**Table 1:** Basic information of the experts (*N* = 20)

Items		N	Items		N
Education background	Bachelor’s Degree	15	Working time	5 ~ 10 years	7
	Master’s degree	1		11 ~ 20 years	10
	Doctor of Medicine	4		21 ~ 30 years	3
Professional title	Medium-grade professional title	15	Region	Hubei Province	15
	Title of a senior professional post	5		Shandong province	3
Age	30 ~ 39	13		Sichuan province	2
	40 ~ 49	2	Research direction	Oncology and palliative care	17
	≥ 50	4		Clinical care	3

### Basic characteristics of the study subjects

Table 2 shows the general characteristics of the hospice care professionals.

**Table 2:** General information of the hospice professionals (*n* = 200)

Items		number	%
Gender	males	19	9.5%
	females	181	90.5%
Age	19 ~ 28	85	42.5%
	29 ~ 38	92	46%
	39 ~ 48	17	8.5%
	≥ 49	6	3%
Educational	Associate degree	37	18.5%
	Bachelor’s degree	152	76%
	Master’s degree	7	3.5%
	Doctoral degree	4	2%
Marital status	Married	125	62.5%
	Unmarried	75	37.5%
Occupation	Nurses	185	92.5%
	Doctors	15	7.5%
Technical titles	Junior professional title	122	61%
	Medium-grade professional title	72	36%
	Title of a senior professional post	6	3%
Working time	2–5 yeas	66	33%
	6–10 yeas	84	42%
	11–20 yeas	36	18%
	>20 yeas	14	7%

### Delphi results

A total of 2 rounds of Delphi method were conducted, 20 scales were distributed in each round, and the effective recovery rate was 100%. In the first round, 10 experts put forward their opinions, and in the second round, two experts put forward their opinions, and the experts were highly motivated. The authority coefficients of the two rounds of experts are 0.838 and 0.833 respectively. The expert authority coefficient of Delphi method is 0.75 ∼ 1. It is generally believed that an expert authority coefficient greater than 0.7 indicates the degree of expert authority [22], so the degree of expert authority in this study is greater. The Kendall consistency of the experts in the first round was 0.121 ∼ 0.200, and the reliability of the experts in the second round ranged from 0.115 to 0.136 (*P* < 0.05).

### Analysis of scale entries

The t values of each item in the high-score group and the low-score group ranged from 5.442 to 10.170 (*P* < 0.05), and there was no item that could be deleted.

### Scale reliability

The reliability of the scale is based on Cronbach’s α coefficient and the half-and-half reliability coefficient, which are commonly used to determine the reliability of the index. It is generally believed that Cronbach’s α coefficient and half-and-half reliability coefficient are greater than 0.7, indicating that the scale has good reliability. (Table 3).

**Table 3:** Reliability of the survey scale for assessing the care burden of hospice healthcare workers

	Number of entries	Cronbach’sα	Spearman-Brown
Work environment Professional roles	9 8	0.920 0.889	0.846 0.817
Clinical nursing	9	0.938	0.891
Psychological burden	7	0.910	0.832
Total	33	0.963	0.927

The Cronbach’s α coefficients of each dimension of the scale were 0.920, 0.889, 0.938 and 0.910 respectively, and the half-and-half reliability coefficients were 0.846, 0.817, 0.891 and 0.832, respectively, while the Cronbach’s α coefficient and half-and-half reliability coefficient of the total scale were 0.963 and 0.927, respectively, all ≥ 0.7, indicating that the scale had good reliability, internal consistency and stability.

#### Validity

##### Content validity (correlation score 1–4)

The validity of the scale was expressed by the content validity index (CVI), including the content validity index of the item level (I-CVI) and the average content validity index of the scale level (S-CVI) [25]. When the I-CVI > 0.78, the content validity at the item level is better [26]. S-CVI/Ave is the average I-CVI for all projects. When the S-CVI/Ave > 0.9, the scale has good content validity at the average level [27].

The I-CVI was 0.90-1 > 0.78, and the content validity at the item level was good. The S-CVI/Ave was 0.967, and the S-CVI/Ave of each dimension was > 0.90, ranging from 0.964 to 0.980. The content validity of the average scale was good.

### Structural validity - exploratory factor analysis

#### KMO and bartlett tests (Table 4)

Table 4 shows that the KMO values are all greater than 0.7, the validity is good, and *P* < 0.001. There is a correlation between variables, so exploratory factor analysis can be carried out.

**Table 4:** KMO and Bartlett tests of the survey scale on the care burden of hospice healthcare workers

	Kaiser-Meyer-Olkin (KMO)	Bartlett test	
		X^2^	P
Total	0.946	5516.160	*P* < 0.001
Work environment	0.910	1184.683	*P* < 0.001
Professional roles	0.870	818.351	*P* < 0.001
Clinical nursing	0.920	1500.484	*P* < 0.001
Psychological burden	0.900	863.750	*P* < 0.001

#### Using principal component maximum variance rotation factor analysis

According to the analysis of the overall structural validity of the scale, the scale has five common factors, and the total cumulative contribution rate is 68.878%. After principal component analysis and maximum orthogonal rotation of variance, the common variance (commensurate) of the scale was more than 0.4, and the factor load of each item was also more than 0.4. Factor 1 is the clinical nursing dimension, factor 2 is the psychological burden dimension, factor 3 and factor 5 are the working environment dimension, and factor 4 is the professional role dimension. It should be noted that the B1 entry in factor 3 is slightly different from the structure of the original scale. However, considering that B1 reflects the content related to professional roles, after expert discussion, the entry remains in the professional role dimension. The specific analysis is shown in Table 5 below. (A is the working environment dimension, B is the professional role dimension, C is the clinical nursing dimension, and D is the psychological burden dimension).

**Table 5:** Factor analysis of maximum variance after orthogonal rotation in the survey scale on the care burden of hospice medical staff

Items	factor 1	factor 2	factor 3	factor 4	factor 5	Communalities
**C5** Do you think that when the personalities of family members and patients are difficult to approach, the implementation of hospice care work will make you feel difficult?	0.723					0.816
**C6** Do you think it is more difficult to handle conflicts between end-stage patients and their families?	0.720					0.819
**C4** Do you think it is difficult to handle patients with depression and those who hold a pessimistic attitude towards death in the work of hospice care?	0.713					0.782
**C3** Do you think it is difficult to communicate with patients who are not aware of their impending death?	0.684					0.733
**C7** Do you think that caring for end-stage patients requires more work (including psychological care and disease care)?	0.591					0.736
**C2** Do you think that when end-stage patients and their families who care for them do not accept the deterioration of their condition, you will feel pressure?	0.577					0.654
**C9** Do you believe that the recognition and cooperation of family members towards hospice care are important factors in carrying out work?	0.574					0.723
**C1** Do you think that the end-stage patients you care for or their families will make excessive demands for care from you?	0.531					0.621
**C8** Do you think that when caring for end-stage patients, they often feel embarrassed due to their demands?	0.495					0.640
**D4** Do you think you feel unprepared when taking care of end-stage children and adolescents?		0.712				0.750
**D6** Do you think it is more difficult to take care of respiratory symptoms in end-stage patients when they are heavily cared for?		0.706				0.718
**D1** Do you think that when caring for end-stage patients, facing their continuous death experiences can make you feel burdened?		0.697				0.712
**D5** Do you think that when the symptoms of end-stage patients cannot be effectively controlled, there will be a sense of powerlessness?		0.690				0.722
**D3** Do you think that in the event of an unexpected situation in end-stage patients (such as sudden cardiac arrest or life-threatening situations) that requires rescue, the burden is heavy?		0.687				0.627
**D2** Do you think it is more difficult to handle the mental symptoms of end-stage patients than the physical symptoms?		0.632				0.668
**D7** Do you think there is insufficient training in symptomatic and psychological care for end-stage patients?		0.599				0.622
**A1** Do you think the uniqueness of the hospice department and the recognition of family and peers are very important?			0.839			0.783
**A2** Do you think that the hospice department needs to further strengthen management and increase economic investment?			0.816			0.853
**A3** Do you think that the hospice department lacks the implementation and participation of government and hospital policies and plans?			0.655			0.680
**B1** Do you think it is possible for you to spend more time caring for end-stage patients?			0.590			0.608
**A4** Do you think that poor communication with colleagues in hospice care can affect the progress of work?			0.580			0.602
**B5** Do you think that in the care work of hospice care, it is difficult to clearly define your professional role?				0.790		0.764
**B8** Do you think that it is more difficult for hospice workers to achieve a sense of professional achievement?				0.732		0.650
**B4** Do you think that the care work of hospice care is quite complex and often requires being on call, which will make your time insufficient (busy work hours and occupying life time)?				0.655		0.672
**B6** Do you think the significance of caring for end-stage patients is reflected in the care process at that time?				0.596		0.593
**B3** Do you think it is necessary to quickly establish a sense of identification with end-stage patients and feel pressure?				0.495		0.624
**B7** Do you think that taking care of end-stage patients requires more emotional investment?				0.432		0.677
**B2** Do you think there is insufficient preparation when dealing with the emotional needs of end-stage patients and their families?				0.423		0.511
**A7** Do you think there is a significant gap between the actual working environment and the ideal for hospice care, resulting in a decrease in expectations and work enthusiasm?					0.723	0.709
**A8** Do you think that in the working atmosphere of hospice care, there is pressure to respond to the high-quality care and quality related requirements of hospitals?					0.685	0.718
**A6** Do you think that excessive exposure to death by the hospice department can cause psychological stress?					0.579	0.582
**A9** Do you think that hospice care currently lacks social recognition and support from other forces in society?					0.563	0.656
**A5** Do you think that when the staffing of the hospice department is insufficient, there is greater pressure at work?					0.486	0.707

## Discussion

### Quality control of scale preparation

In the process of developing the scale, we first consulted a large number of related studies at home and abroad under the guidance of the Zarit Nursing Burden Scale (ZBI) to ensure the standardization, rigor and rationality of the scale. After 2 rounds of Delphi method, the relevant items of the scale were further revised. We selected experts in the fields of clinical nursing, geriatric nursing, nursing management, nursing education, nursing research, oncology clinics, etc., and proposed constructive suggestions for the revision of the contents of the scale to ensure its quality. In the process of sending the scale to the expert, we carefully checked whether there were missing items in each scale to ensure the effectiveness of the scale collection. After 2 rounds of Delphi method, the effective recovery rate of the scale was 100%. In the first round, 10 experts put forward their opinions, and in the second round, 2 experts put forward their opinions. The authority coefficients of the two rounds of experts are 0.838 and 0.833, respectively, indicating a high degree of authority. Kendall’s W coefficient of the first-round expert opinion test was 0.121-0.200 (*P* < 0.05), and Kendall’s W coefficient of the second-round expert opinion test was 0.115–0.136 (*P* < 0.05).

### Reliability evaluation of the scale

In terms of reliability, it is generally believed that the reliability of a scale is good when the Cronbach’s α coefficient and Spearman-Brown coefficient are above 0.7. The Cronbach’s α coefficients of each dimension of the scale are 0.920, 0.889, 0.938, 0.910, 0.86, 0.817, 0.891, 0.832 and 0.927, indicating that the reliability, internal consistency and stability of the scale are good.

### Validity evaluation of the scale

#### Content validity

Content validity, also known as apparent validity or logical validity, refers to whether each item of the scale measures what it wants to measure, that is, whether the object’s understanding and answer to the question is consistent with what the item designer wants to ask [28]. In this study, the Delphi method was used to invite experts to score the relevance of the scale and evaluate its content validity. When I-CVI/Ave > 0.78 and S-CVI/Ave > 0.9, the content validity of the scale is good. According to the results of expert evaluation, the item-level content validity (I-CVI) is 0.90-1.00, and the average scale-level content validity (S-CVI) of the total scale is 0.967, indicating that the scale has good content validity.

#### Structural validity

Construct validity, also known as construct validity or feature validity, refers to whether the structure of the scale is consistent with the theoretical hypothesis of tabulation and whether the internal components of the measurement results are consistent with the field that the designer intends to measure; the commonly used statistical method is factor analysis, which reflects the contribution of a project to the field. The greater the factor load value is, the closer the relationship is to the domain [29]. Five common factors were extracted based on a characteristic root > 1, which explained 68.878% of the total variation. The commonness of 33 items in the scale is ≥ 0.4, and the factor load of each item is also ≥ 0.4, indicating that the construct validity of the scale is good.

#### The practicality and significance of the scale

On the basis of an extensive literature review and Delphi method, the nursing burden scale of hospice care professionals in China was developed. To clarify the current situation and influencing factors of the care burden of hospice care professionals in China, and to evaluate the effect of intervention measures on the care burden of hospice care professionals. At present, hospice care has received increasing attention, and a series of problems have emerged. One of the problems related to health care staff is the nursing burden. The scale developed in this study is practical and helpful for nursing managers to formulate intervention measures to reduce their nursing burden and improve the efficiency of hospice care.

#### Limitations and further research

As with any study, this study had several important limitations. In this study, exploratory factor analysis was used to develop and verify the scale, which ensured the scientific nature of the study in terms of methodology. However, in the actual investigation process, because there are many nurses involved in hospice care in the oncology department, most of the population was selected from the oncology department, which may have biased the results. There are 33 items in total. In the future, a short version of the scale will be further developed and verified in multiple centers to ensure the popularization of the scale.

## Conclusions

The reliability and validity test showed that the care burden scale of hospice care professionals developed in this study has good reliability and validity and can be used to evaluate the level of care burden of hospice care professionals in China. However, confirmatory factor analysis was not performed for the scale, and the selected samples were mainly medical staff engaged in or carrying out hospice care pilot institutions in Hubei Province. The representativeness of the sample size needs to be studied, and the sample size will be further expanded in multiple centers to improve the content of the scale.

### Electronic supplementary material

Below is the link to the electronic supplementary material.

Supplementary Material 1

## Data Availability

The datasets used and/or analysed during the current study are available from the corresponding author upon reasonable request.
