# Supplementary material for: Development and validation of the hospice professional coping scale among Chinese nurses
Source: BMC Health Serv Res. 2024 Apr 20;24:491. doi: 10.1186/s12913-024-10970-9 (PMC11031935; doi:10.1186/s12913-024-10970-9)
Supplement: Supplementary file 1 — Supplementary Material 1 [file 12913_2024_10970_MOESM1_ESM.docx]

**Supplementary table 1 Concentration of expert opinions (First round)**

| dimensionality | Items | Concentration index of expert opinions | | |
| --- | --- | --- | --- | --- |
|  |  | Importance score$\bar{x}$+s | CV | Kj |
| 1. Work environment | 01 Do you think that the particularity of the hospice care sector will be isolated by peers? | 4.00±0.918 | 0.229 | 0.35 |
|  | 02 Do you think there is a lack of management and financial support for the hospice care sector? | 4.30±0.923 | 0.214 | 0.55 |
|  | 03 Do you think there is a lack of engagement in the palliative care sector? | 4.15±0.933 | 0.225 | 0.45 |
|  | 04 Do you think that poor communication with colleagues in hospice care affects your work? | 4.00±0.795 | 0.199 | 0.25 |
|  | 05 Do you think that the hospice sector does not have enough outside information? | 3.65±0.933 | 0.256 | 0.20 |
|  | 06 Do you think that there is more stress at work in hospice care departments when there is not enough staff? | 4.40±0.754 | 0.171 | 0.55 |
|  | 07 Do you think that excessive exposure to death in hospice care causes psychological stress? | 4.05±0.945 | 0.233 | 0.40 |
|  | 08 Do you think that there is a big gap between the actual working environment of hospice care and the ideal one, and you will feel a sense of loss? | 4.00±0.795 | 0.199 | 0.25 |
|  | 09 Do you think it is stressful to cope with the quality of care and quality related requirements in the hospice work environment? | 3.70±0.923 | 0.249 | 0.20 |
| 2. Professional roles | 01 Do you think it's possible to spend more time caring for terminally ill patients? | 4.10±0.641 | 0.156 | 0.25 |
|  | 02 Do you believe that there is insufficient preparation to deal with the emotional needs of end-stage patients and their families? | 3.80±0.834 | 0.219 | 0.20 |
|  | 03 Do you think that you often feel the pressure to quickly establish a sense of identity with end-stage patients? | 4.20±0.834 | 0.198 | 0.45 |
|  | 04 Do you think that the complexity of hospice care and the need to be on call often leave you with insufficient time (working time and living time)? | 3.95±0.945 | 0.239 | 0.35 |
|  | 05 Do you think it is difficult to define your professional role in hospice care? | 4.15±0.933 | 0.225 | 0.45 |
|  | 06 Do you think that the meaning of caring for patients at the end of life is reflected in the process of care? | 3.95±0.887 | 0.225 | 0.25 |
|  | 07 Do you think caring for terminally ill patients is too emotionally demanding? | 3.85±0.988 | 0.256 | 0.25 |
| 3. Clinical care | 01 Do you think that the terminally ill patients you care for or their families make too many demands on you? | 3.50±0.889 | 0.231 | 0.15 |
|  | 02 Do you think that you feel stressed when caring for end-stage patients and families who do not accept deterioration? | 4.05±0.945 | 0.23 | 0.40 |
|  | 03 Do you think it is difficult to communicate with patients who do not know they are dying? | 3.95±0.945 | 0.239 | 0.35 |
|  | 04 Do you think that it is difficult to deal with patients with depression and those with a strong identity with death in hospice care? | 4.00±0.973 | 0.243 | 0.40 |
|  | 05 Do you think that it is difficult to carry out hospice care when the family and the patient's personality are difficult to relate to? | 4.20±0.894 | 0.213 | 0.45 |
|  | 06 Do you think that when terminally ill patients have deep conflicts with their families, you find it more difficult to deal with them? | 4.25±0.786 | 0.185 | 0.45 |
|  | 07 Do you think more needs to be done to care for patients at the end of life (including psychological comfort, disease care, emotional needs)? | 4.45±0.759 | 0.171 | 0.60 |
|  | 08 Do you think that terminally ill patients only look forward to your care and you seem to be the only person they can rely on? | 3.60±0.995 | 0.276 | 0.20 |
|  | 09 Do you think caring for terminally ill patients is often difficult because of the demands they make? | 3.60±0.883 | 0.245 | 0.20 |
| 4. Psychological burden | 01 Do you find caring for patients at the end of life burdensome when facing their repeated death experiences? | 4.20±1.005 | 0.239 | 0.55 |
|  | 02 Do you think that dealing with the mental symptoms of terminally ill patients is more difficult than the physical symptoms? | 4.15±0.875 | 0.211 | 0.40 |
|  | 03 Do you think that terminally ill patients have a heavy burden when they need to be rescued in the event of an accident? | 3.90±0.912 | 0.234 | 0.30 |
|  | 04 Do you think you feel ill-prepared to care for children and adolescents at the end of life? | 4.15±0.988 | 0.238 | 0.50 |
|  | 05 Do you think there is a sense of powerlessness when the symptoms of end-stage patients are not effectively controlled? | 4.05±0.945 | 0.233 | 0.40 |
|  | 06 Do you think that it is more difficult to care for patients with severe respiratory symptoms at the end stage? | 4.15±0.875 | 0.211 | 0.40 |
|  | 07 Do you think there is not enough training in the symptomatic care of patients at the end of their life? | 3.90±0.968 | 0.248 | 0.35 |

After the first round of expert consultation, the items were added or modified according to the scores of experts on the importance and relevance of the items and the textual opinions of experts. Results were as follows:

(1) Three items were deleted because of their coefficient of variation > 0.25 (two items were deleted and one item was retained) as follows:

Dimension of work environment: 05 Do you think that the hospice care department does not have enough external information?

Professional role dimension: 07 Do you think that caring for terminal patients requires too much emotional demand? (After discussion with the group, this item is retained considering its importance and the coefficient of variation is not very high.)

Do you think that patients at the end of life only look forward to your care, and you seem to be the only person he can rely on?

(2) One item was deleted because the full score ratio was less than 0.2, as follows:

Dimensions of clinical care: 01 Do you think that the terminally ill patients you care for or their families ask too much from you? (After discussion with the group, this item is retained in consideration of its importance.)

(3) Three new entries are added as follows:

A new item was added in the dimension of work environment: Do you think that palliative care currently lacks social recognition and support from other social forces?

A new item was added to the professional role dimension: Do you think it is more difficult for hospice care staff to achieve a sense of professional accomplishment?

A new item was added in the dimension of clinical care: Do you think that family's recognition and cooperation with palliative care is an important factor in the implementation of work?

(4) Ten items were revised descriptively as follows:

Dimensions of working environment:

"01 Do you think that the specificity of the hospice care sector will isolate you from your peers?" "Colleagues" is replaced by "family or colleagues";

"02 Do you think there is a lack of regulatory and financial support for the palliative care sector?" "Do you think there is a need for better management and financial investment in the palliative care sector?" ;

"03 Do you think there is a lack of involvement in the palliative care sector?" "Do you think there is a lack of implementation and participation of government and hospital policies and plans in the palliative care sector?" ;

"04 Do you think that when you work with colleagues in hospice care, poor communication affects your work?" 04 Do you think that poor communication with colleagues in hospice care affects the performance of your work?

"08 Do you think that there is a big gap between the actual working environment of hospice care and the ideal one, and you will feel a sense of loss?" Do you think that there is a big gap between the real work environment and the ideal work environment in palliative care, and the expectations and enthusiasm for work are low? ;

In the question "Do you think that it is stressful to cope with the quality care and quality related requirements of the hospital in the work atmosphere of hospice care", "coping" was replaced with "coping".

Dimensions of professional roles:

"04 Do you think that the complexity of hospice care and the need to be on call often make your time (working time and living time) insufficient?" Do you think that the complexity of hospice care and the need to be on call often make your time insufficient (busy working hours and life time)?

"07 Do you think that caring for terminally ill patients is too emotionally demanding? "Too much" is replaced by "more".

Dimensions of clinical nursing:

"Do you THINK THAT more needs to be done (including psychological comfort, disease care, emotional needs) in caring for patients at the end of life?" "Do you think more needs to be done (including psychological care, disease care) to care for patients at the end of life?"

Dimensions of psychological burden:

"03 Do you think that terminally ill patients have a heavy burden when they need to be rescued by accident?" 03 Do you think that terminally ill patients have a heavy burden when they need to be rescued in the event of an unexpected event (such as sudden cardiac arrest, which is life threatening)?

**Supplementary table 2 Concentration of expert opinions (Second round)**

| dimensionality | Items | Concentration index of expert opinions | | |
| --- | --- | --- | --- | --- |
|  |  | Importance score$\bar{x}$+s | CV | Kj |
| 1. Work environment | 01 Do you think that the particularity of the hospice care sector leads to isolation from your family or colleagues? | 4.35±0.745 | 0.171 | 0.50 |
|  | 02 Do you think the palliative care sector needs to be further regulated and financially invested in? | 4.40±0.598 | 0.136 | 0.45 |
|  | 03 Do you think there is a lack of implementation and participation of government and hospital departments in the palliative care sector? | 4.50±0.761 | 0.169 | 0.60 |
|  | 04 Do you think that poor communication with colleagues in hospice care affects the performance of your work? | 3.95±0.826 | 0.209 | 0.25 |
|  | 05 Do you think that there is more stress at work in hospice care departments when there is not enough staff? | 4.15±0.745 | 0.180 | 0.30 |
|  | 06 Do you think that excessive exposure to death in hospice care causes psychological stress? | 4.00±0.918 | 0.230 | 0.30 |
|  | 07 Do you think that there is a big gap between the real work environment and the ideal work environment in palliative care, and the expectations and work enthusiasm are low? | 3.90±0.852 | 0.218 | 0.25 |
|  | 08 Do you think it is stressful to cope with the quality of care and quality related requirements in the hospice work environment? | 4.00±0.795 | 0.199 | 0.30 |
|  | 09 Do you think there is a lack of social recognition and support for palliative care? | 4.20±0.616 | 0.147 | 0.30 |
| 2. Professional roles | 01 Do you think it's possible to spend more time caring for terminally ill patients? | 3.95±0.826 | 0.209 | 0.25 |
|  | 02 Do you believe that there is insufficient preparation to deal with the emotional needs of end-stage patients and their families? | 4.00±0.725 | 0.181 | 0.25 |
|  | 03 Do you think that you often feel the pressure to quickly establish a sense of identity with end-stage patients? | 4.10±0.852 | 0.208 | 0.35 |
|  | 04 Do you think that the complexity of hospice care and the need to be on call often leave you short of time (busy work hours and life time)? | 4.05±0.945 | 0.233 | 0.35 |
|  | 05 Do you think it is difficult to define your professional role in hospice care? | 3.70±0.923 | 0.249 | 0.20 |
|  | 06 Do you think that the meaning of caring for patients at the end of life is reflected in the process of care? | 4.00±0.795 | 0.199 | 0.30 |
|  | 07 Do you think caring for patients at the end of life is more emotionally demanding? | 3.95±0.826 | 0.209 | 0.30 |
|  | 08 Do you think that it is more difficult for hospice staff to achieve a sense of professional fulfillment? | 4.15±0.875 | 0.211 | 0.40 |
| 3. Clinical care | 01 Do you think that the terminally ill patients you care for or their families make too many demands on you? | 3.90±0.912 | 0.234 | 0.30 |
|  | 02 Do you think that you feel stressed when caring for end-stage patients and families who do not accept deterioration? | 4.00±0.795 | 0.176 | 0.25 |
|  | 03 Do you think it is difficult to communicate with patients who do not know they are dying? | 4.05±0.826 | 0.204 | 0.30 |
|  | 04 Do you think that it is difficult to deal with patients with depression and those with a strong identity with death in hospice care? | 4.10±0.788 | 0.192 | 0.35 |
|  | 05 Do you think that it is difficult to carry out hospice care when the family and the patient's personality are difficult to relate to? | 4.10±0.788 | 0.192 | 0.35 |
|  | 06 Do you think that when terminally ill patients have deep conflicts with their families, you find it more difficult to deal with them? | 4.15±0.875 | 0.211 | 0.40 |
|  | 07 Do you think that more needs to be done (including psychological care, disease care) in caring for patients at the end of life? | 4.25±0.851 | 0.200 | 0.45 |
|  | 08 Do you think caring for terminally ill patients is often difficult because of the demands they make? | 3.70±0.923 | 0.249 | 0.25 |
|  | 09 Do you think that the family's acceptance and cooperation with hospice care is an important factor in the implementation of hospice care? | 4.35±0.745 | 0.171 | 0.45 |
| 4. Psychological burden | 01 Do you find caring for patients at the end of life burdensome when facing their repeated death experiences? | 3.85±0.813 | 0.211 | 0.25 |
|  | 02 Do you think that dealing with the mental symptoms of terminally ill patients is more difficult than the physical symptoms? | 4.05±0.826 | 0.204 | 0.30 |
|  | 03 Do you think that terminally ill patients have a heavy burden when they need to be rescued in the event of an unexpected event, such as a sudden cardiac arrest, which is life threatening? | 3.90±0.912 | 0.234 | 0.30 |
|  | 04 Do you think you feel ill-prepared to care for children and adolescents at the end of life? | 3.90±0.852 | 0.218 | 0.25 |
|  | 05 Do you think there is a sense of powerlessness when the symptoms of end-stage patients are not effectively controlled? | 3.90±0.912 | 0.234 | 0.30 |
|  | 06 Do you think that it is more difficult to care for patients with severe respiratory symptoms at the end stage? | 4.15±0.933 | 0.225 | 0.45 |
|  | 07 Do you think there is not enough training in the symptomatic care of patients at the end of their life? | 4.15±0.933 | 0.225 | 0.40 |

After the second round of expert consultation, the items were revised according to the scores of experts on the importance and relevance of the items and the textual opinions of experts. Results were as follows:

In this round of expert consultation, according to the conditions of mean (Mj), coefficient of variation (CV) and full score ratio (Kj), there was no item to be deleted in this round, and the conditions were met.

According to expert opinion, one item has been revised:

In the clinical care dimension, "04 Do you think it is difficult to deal with patients with depression and those who have a strong identity with death in hospice care?" "Patients with a strong identification with death" was changed to "patients with a pessimistic attitude towards death".
